# Classification of juvenile spondyloarthropaties according to asas criteria

**DOI:** 10.1186/1546-0096-12-S1-O10

**Published:** 2014-09-17

**Authors:** María M Katsicas, Ricardo A Russo

**Affiliations:** 1 Immunology & Rheumatology, Hospital JP Garrahan, Buenos Aires, Argentina

## Introduction

The juvenile spondyloarthropathies (JSpA) are a group of related seronegative rheumatic diseases characterized by involvement of the axial joints, peripheral large joints and entheses. JSpA and adult SpA are probably parts of the same disease continuum. JSpA are represented by enthesitis related arthritis (ERA). juvenile psoriatic arthritis (JPsA) and probably undifferentiated arthritis (UA) in the ILAR criteria. Sets of classification criteria have been developed in adult patients with SpA. The ASAS classification criteria for axial and peripheral SpA have not been validated in pediatric populations.

## Objectives

To assess the sensibility and specificity of the ASAS criteria for identification of patients with JSpA (ERA, JPsA and UA) among the different Juvenile idiopathic arthritis (JIA) categories. To compare the performance of the ASAS criteria with that of ESSG classification criteria in JIA patients. To identify associations between criteria fulfillment and disease features.

## Methods

Consecutive patients with JSpA followed in our center with complete records were included. Clinical charts and databases were retrospectively reviewed. Randomly selected patients with oligoarthritis, polyarthritis RF negative and systemic arthritis from our cohort served as controls. Demographic and clinical characteristics as well as disease duration at first visit and follow up time were recorded. Items corresponding to the ASAS, ESSG, Amor, seronegative enthesopathy and arthropathy (SEA) syndrome and Modified New York (NY) criteria were obtained from first visit and during disease course. Descriptive , summary statistics (sensitivity [sen], specificity [sp], positive predictive value [PPV] and negative predictive value [NPV]) and Wilcoxon Rank sum test were used.

## Results

106 patients with JSpA (103 ERA, 2JPsA, 1UA) were included (M:92), age at onset: 10 (1-15) years, disease duration at first visit 10 (1-15) months, follow-up time 4 (1-12) years. Controls: 65 patients with other JIA (24 oligoarthritis, 21 polyarthritis RF negative, 20 systemic) (M: 27). At first visit cases showed: 103 (97%) arthritis, 87 (82%) asymetrical oligoarthritis, 67 (63%) elevated CRP, 52 (49%) limitation of lumbar spine motion, 51 (48%) HLA-B27, 45 (42%) enthesitis, 39 (37%) low back pain, 32 (30%) good response to NSAIDS, 26 (25%) positive family history, 20 (19%) radiographic bilateral sacroiliitis grade 2-4, 13 (12%) dactylitis, 8 (8%) uveitis, 7 (7%) unilateral sacroiliitis grade 3-4, 7 (7%) diarrhea, 5 (5%) previous infectious disease, 2 (2%) urethritis, 2 (2%) inflammatory bowel disease, 2 (2%) buttock pain, 1 (1%) psoriasis. At first visit (106 patients): 79 (75%) patients fulfilled ASAS criteria, 78 (74%) peripheral ASAS, 78 (74%) ESSG, 69 (65%) Amor (58 definite, 11 probable), 32 (30%) SEA, 25 (24%) NY (22 definite, 3 probable), 24 (23%) axial ASAS. During disease course (102 patients): 100 (98%) patients fulfilled ESSG criteria, 97 (95%) ASAS, 97 (95%) peripheral ASAS, 94 (92%) Amor (82 definite, 12 probable), 75 (74%) NY (63 definite, 12 probable), 42 (41%) SEA, 41 (40%) axial ASAS. ASAS sen 95%, sp 83%, PPV 90%, NPV92%. ESSG sen 98% sp 89% PPV 93%, NPV 97%. Unilateral and bilateral sacroiliitis were associated only with axial ASAS (p=0,0066 and 0,04 respectively).

## Conclusion

In our cohort ASAS criteria performed similarly to ESSG criteria in the classification of JSpA. Both sets of criteria allow the inclusion of JSpA patients under one unified category to facilitate research and communication. Peripheral ASAS criteria allow the classification of all patients with JSpA who meet axial ASAS criteria.

## Disclosure of interest

None declared.

